# Diagnosis with Metagenomic Next-Generation Sequencing (mNGS) technology and real-time PCR for SARS-CoV-2 Omicron detection using various nasopharyngeal swabs in SARS-CoV-2 Omicron

**DOI:** 10.1371/journal.pone.0305289

**Published:** 2024-08-06

**Authors:** Sujuan Li, Yuanhang Zhang, Pengcheng Tong, Wei Yang

**Affiliations:** Department of Clinical Laboratory, Hangzhou Traditional Chinese Medicine Hospital Affiliated to Zhejiang Chinese Medical University, Hangzhou, Zhejiang, China; Nigerian Institute of Medical Research, NIGERIA

## Abstract

**Background:**

The SARS-CoV-2 Omicron variant, with the main subtypes BA.5.2 and BF.7 in China, led to off-target effects on the S and N genes from December 1, 2022, to January 31, 2023. The kits used for studying and developing these agents were not adequately and independently evaluated. It is important to verify the performance of commercial Real-Time quantitative PCR (RT-qPCR) tests.

**Objective:**

We conducted a clinical evaluation of two Real Time SARS-CoV-2 Omicron assays to verify their performance using various detection reagents and clinical specimens.

**Methods:**

We performed clinical evaluations of two existing Chinese SARS-CoV-2 Omicron RT-qPCR kits 2019-nCoV nucleic acid diagnostic kits (Fosun Biotechnology, National instrument registration 20203400299, Shanghai, China) and COVID-19 nucleic acid detection kits (eDiagnosis Biomedicine, National instrument registration 20203400212, Wuhan, China) and using BSD (Bondson) (Guangzhou Bondson Biotechnology Co. Ltd, batch number 2022101), quality controls provided by the inspection center and a large number of clinically confirmed specimens.

**Results:**

The concordance rates for the Fosun and eDiagnosis kits were 95% and 100%, respectively. The detection limit for the Fosun and eDiagnosis kits was verified to be 300 copies/mL and 500 copies/mL. The Fosun assay exhibited the largest coefficient of variation (CV) for ORF1ab and N gene at the detection limit concentration (4.80%, 3.49%), whereas eDiagnosis showed a smaller CV (0.93%, 1.10%). In the reference product from the Hangzhou Clinical Laboratory Center test, it was found that Fosun had the lowest sensitivity of 93.47% and a specificity of 100%, while eDiagnosis exhibited 100% for both sensitivity and specificity. The lowest single target gene detection rate of Fosun reagents was 68.7% for the ORF1ab gene and 87.5% for the N gene, while eDiagnosis detection rate was 100%. Among the clinical group S specimens, the missed detection rate of the Fosun reagent was 10.9%, which was higher than the 3.9% of eDiagnosis. However, there was no significant difference in the clinical diagnostic efficiency of the two reagents.

**Conclusions:**

The ORF1ab and N assays of SARS-CoV-2 Omicron on the eDiagnosis platform yielded higher values compared to those on the Fosun platform. Consequently, the eDiagnosis kit has also been used as standard detection reagents. Considering that the Fosun reagent has a relatively low detection limit and targets three single genes, it is more advantageous as a confirmatory reagent for the new museum.

## 1. Introduction

A new variant of concern (VoC) of SARS-CoV-2, known as Omicron, has emerged in a world weary from the COVID-19 pandemic, resulting in significant negative impacts on health, social interactions, and economic stability [1]. Rapid detection of the SARS-CoV-2 Omicron variant in a patient’s respiratory tract sample may influence treatment options, as this variant is known to evade certain monoclonal antibodies [2]. In terms of diagnostic technology, the SARS-CoV-2 Omicron variant can be detected using Real-Time quantitative PCR (RT-qPCR) platforms, which are widely used in South Africa [3]. There is no reason to believe that current treatment regimens and therapies for the SARS-CoV-2 Omicron variant will no longer be effective, except possibly for monoclonal antibodies [4]. However, it is important to note that there is currently a significant amount of data on susceptibility to the SARS-CoV-2 Omicron variant [5].

The open reading frame (ORF), nucleocapsid (N) genes, and envelope (E) genes of the coronavirus are the most frequently targeted regions for detecting the SARS-CoV-2 Omicron variant [6]. RT-qPCR can detect the amount of viral RNA in real time and is considered the gold standard for detecting SARS-CoV-2 Omicron [7]. In routine clinical practice, it is common to encounter some clinical tests that yield unqualified and false-positive results [8]. These inaccuracies have a significant negative impact on the effectiveness of epidemic prevention and control efforts [9–11].

In this study, the RT-qPCR assay for SARS-CoV-2 Omicron testing was validated using three methods. These methods included the use of a new liquid performance verification RT-qPCR reference product for SARS-CoV-2 Omicron RNA (coronavirus ribonucleic acid) from Guangzhou Bondson Biotechnology Co., Ltd. in Guangzhou, China. Additionally, RNA pseudovirus samples were obtained from the Clinical Laboratory Center of the National Health Commission and the Clinical Laboratory Center in Hangzhou. Lastly, specimens were collected from individuals with symptomatic infections or patients who underwent testing voluntarily. These specimens were specifically nasopharyngeal swab (NPS) specimens, collected from clinically confirmed positive patients. We evaluated the performance of two commercial RT-qPCR diagnostic kits for SARS-CoV-2 Omicron. The main verification parameters included analytical accuracy, sensitivity, specificity, and clinical validation. This study provides a reference for clinical laboratories to use when selecting SARS-CoV-2 Omicron nucleic acid detection kits.

## 2. Materials and methods

### 2.1 Materials

#### 2.1.1 Performance verification reference product

We used the SARS-CoV-2 Omicron RNA liquid performance verification reference product BDS (Bondson) from Guangzhou Bondson Biotechnology Co. Ltd. The batch number is 2022101. This product is based on the SARS-CoV-2 Omicron pseudovirus culture medium and contains the essential genes of SARS-CoV-2 Omicron, including the full-length N, E, and ORF1ab genes. The reference product is uniform, stable, and has good interoperability with clinical samples because it utilizes droplet digital PCR (ddPCR) combined with fluorescence quantitative PCR.

#### 2.1.2 Hangzhou Clinical Laboratory Center quality control performance verification reference product

We used the one-year reference product from the Hangzhou Clinical Laboratory Center, which consisted of 12 groups of quality control products. Each group consisted of five specimens, with serial numbers ranging from 22011 to 22125. The groups were labeled as follows: G1-G5, G6-G10, G11-G15, G16-G20, G21-G25, G26-G30, G31-G35, G36-G40, G41-G45, G46-G50, G51-G55, and G56-G60. In 2022, there were 10 reference samples from the National Health Commission Clinical Laboratory Center, labeled S1-S10, and 10 patientsamples from previous tests, labeled S11-S20, totaling 20 samples.

#### 2.1.3 Patients and samples

We collected 504 nasopharyngeal swabs from individuals at Hangzhou TCM Hospital, which is affiliated with Zhejiang Chinese Medicine University. We provided individuals with four sets of samples, including positive and negative specimens, categorized as Group S, 10-tube sampled specimens (10-group), 20-tube sampled specimens (20-group), and the control Group N. The S group consisted of 183 single-sampled specimens. In the 10-group, there were 46 specimens with 10 mixed samples. In the 20-group, there were 225 specimens with 20 mixed samples. The N-group consisted of 50 specimens from individuals with normal conditions.

#### 2.1.4 SARS-CoV-2 Omicron diagnostic kits

Two commercial RT-qPCR diagnostic test kits are available for the detection of Novel Coronavirus 2019-nCoV nucleic acid using the RT-qPCR method for SARS-CoV-2 Omicron. The Fosun kit (Diagnostic Technology Co. Ltd., Shanghai, China; batch number C20220744, National instrument registration 20203400299) and the eDiagnosis kit (Wuhan Easy Diagnosis Biomedicine Co. Ltd., Wuhan, China; batch number 2022, National instrument registration 20203400212) were used in this study. Basic information on the RT-qPCR diagnostic kits for SARS-CoV-2 Omicron is presented in Table 1.

**Table 1:** Under the condition that the collected specimens are qualified, the positive and negative values of the two reagents are significantly different, as shown in the figure below.

Detection reagents	eDiagnosis kit		Fosun kit		
Fluorescence channels	FAM	HEX	FAM	VIC	ROX
	(N gene)	(ORF1ab)	(ORF1ab)	(N gene)	(E gene)
Positive verdict	FAM channel amplification curve increases exponentially and Ct <38 is FAM positive. HEX channel amplification curve increases exponentially and Ct <38 is HEX positive.		FAM has an amplified signal, Ct ≤36, and the expansion curve is typically S-shaped, then ORF1ab (+). VIC has an amplification signal, Ct ≤36, and the expansion curve is typically S-shaped, then N gene (+). ROX has an amplification signal, Ct ≤36, and the expansion curve is typically S-shaped, then the E gene (+).		
Negative decision value	FAM channel amplification curve has no exponential growth or Ct>40 is FAM negative. HEX channel amplification curve has no exponential growth or Ct>40 is HEX negative.		FAM channel amplification curve has no exponential growth or Ct>36 is FAM negative. VIC channel amplification curve has no exponential growth or Ct>36 is VIC negative. ROX channel amplification curve has no exponential growth or Ct>36 is ROX negative		

#### 2.1.5 Nucleic acid instrumentation

The Stream MD-NAS96, an automatic nucleic acid extraction instrument developed by eDiagnosis Gene Co. Ltd. in Wuhan, and the Fosun F-AutoFex96 automatic nucleic acid extractor, were used for the extraction of nucleic acids. RT-qPCR was conducted using the Yarui MA-600 real-time PCR system (Suzhou Yarui Biotechnology Co. Ltd., Suzhou, China).

### 2.2. Methods

#### 2.2.1 SARS-CoV-2 Omicron genotyping by mNGS

The nasopharyngeal swab was inactivated using AVL buffer containing guanidine salts (Vision Medicals, China) and RNA extraction was performed (Vision Medicals, China). Libraries were prepared using the Vision Medicals (China) Novel Coronavirus (2019-nCoV) Assay, 300 mL of sample was taken, followed by 50 bp single-ended sequencing using Illumina Nextseq 487.High-throughput sequencing libraries are often combined in batches, where multiple samples are mixed, and a chip is added. After sequencing, it is necessary to differentiate between different samples by identifying the labels on the sequences, known as barcodes, which are typically 6~10 base pairs (bp) are artificially synthesized sequences with known sequences. The removal of low-quality reads, adaptor sequences, repeated reads, and those shorter than 50 base pairs in the raw data is performed using Trimmomatic [12]. Low-complexity reads were filtered by Kcomplexity using default parameters. Human sequence data were identified and excluded by mapping to a human reference genome (GRCh38) using SNAP v1.0beta.18. Microbial reads were aligned to the database using the Burrows-Wheeler Aligner software tool. To construct the microbial genome database, pathogens and their genomes or assemblies were selected based on the Kraken2 criteria for choosing representative assemblies for microorganisms, including bacteria, viruses, fungi, protozoa, and other multicellular eukaryotic pathogens. The non-human high-quality sequences were compared with the microbial database to obtain species annotation results and generate the detection report [13–15].

#### 2.2.2 Real-time RT-qPCR assays for SARS-CoV-2 Omicron RNA detection

The eDiagnosis (Wuhan Easy Diagnosis Biomedicine Co. Ltd., Wuhan, China) utilized the Novel Coronavirus 2019-nCoV Nucleic Acid Detection Kit-Fluorescent PCR method, in accordance with the instructions provided by the manufacturer. Each 20μL reaction mixture contained N gene primers/probes, ORF1ab primers/probes, internal standard primers/probes, DNA polymerase, reverse transcriptase, UNG, and RNase inhibitors. DNTPs serve as the template. RT-qPCR was performed on a Yarui MA-6000 real-time PCR system (Suzhou Yarui Biotechnology Co. Ltd., Suzhou, China). The thermal cycling conditions were as follows: 50°C for 15 minutes, 95°C for 30 seconds, followed by 40 cycles of 95°C for 3 seconds and 60°C for 40 seconds. The signals from the FAM, VIC, and ROX fluorescence channels were acquired at 60°C.The other reagent used was the Fosun diagnostic reagent, which utilized the Novel Coronavirus 2019-nCoV nucleic acid detection kit with the fluorescent PCR method, following the manufacturer’s instructions. The reagents consisted of the 2019-nCoV reaction solution and PCR enzyme. Each specimen was mixed with 20 μl of reaction solution, 6 μl of enzyme, and 14 μl of the reaction solution containing the enzyme. RT-qPCR was performed on a Yarui MA-6000 real-time PCR system (Suzhou Yarui Biotechnology Co., Ltd., Suzhou, China). The thermal cycling conditions were as follows: 50°C for 15 minutes, 95°C for 3 minutes, and 95°C for 5 seconds. This was followed by five cycles where signals from the FAM, JOE, ROX, and CY5 fluorescence channels were not recorded. After that, there were 40 cycles of 95°C for 5 seconds and 60°C for 40 seconds. During these cycles, signals from the FAM, JOE, ROX, and CY5 fluorescence channels were acquired at 60°C.

RT-qPCR cycle parameters were set according to the kit instructions. The baseline was usually set automatically by the instrument. Baseline adjustment principles include selecting regions with stable fluorescence signals prior to exponential amplification, avoiding signal fluctuations at the beginning of fluorescence acquisition, and reducing the threshold period (Cp)/quantification period (Cq) value of the sample with the earliest exponential amplification by 1-2 cycles at the end. However, RT-qPCR amplification conditions of reagents in two were different, and there was a significant difference between the predicted positive and negative values.

#### 2.2.3 Coincidence rate verification

In 2022, there were 10 reference samples from the National Health Commission Clinical Laboratory Center, labeled S1–S10, and 10 patient samples from previous tests, labeled S11–S20, making a total of 20 samples. Nucleic acid extraction was performed using the Fosun and eDiagnosis nucleic acid extraction kits. Amplification was performed using Fosun (Diagnostic Technology Co. Ltd., Shanghai, China; batch number C20220744, National instrument registration 20203400299) and eDiagnosis (Wuhan Easy Diagnosis Biomedicine Co. Ltd., Wuhan, China; batch number 2022, National instrument registration 20203400212) kits for detecting SARS-CoV-2 Omicron, using the same Yarui MA-6000 real-time PCR system. A kit was considered to have passed verification if the coincidence rate of negative and positive results was ≥ 95%.

#### 2.2.4 Limit of detection

The concentrations of the limit of detection (LOD) reference products L1–L5 provided in the BDS were 13,900, 2,780, 927, 309, and 103 copies/mL. Separately, each sample was tested 20 times, and the Limit of Detection (LOD) was initially screened at the lowest concentration where 20 results were positive. Each sample was then subjected to 20 consecutive Limit of Detection (LOD) assays in different batches (five batches in total, with four repeated tests per batch) for statistical analysis. At the lowest concentration level, the detection rate is 100% positive due to the Limit of Detection (LOD) of each kit (CNAS-CL02-A0009).

#### 2.2.5 Precision

In the first place, the precision reference products in BDS include low-concentration products (eDiagnosis 500 copies/mL) and (Fosun 300 copies/mL). Two commercially available SARS-CoV-2 Omicron diagnostic kits were used for amplification in the same instrument. Measure one batch per day continuously for 4 days, and repeat the measurement 5 times for each batch. Therefore, 20 measurements were taken at each concentration. When the coefficient of variation (CV) precision value is less than 5%, the precision verification is considered to have passed.

#### 2.2.6 Cross contamination and specificity

Specific verification requires selecting virus samples with infection sites or symptoms similar to the new coronavirus for testing. SARS-CoV-2 Omicron, HCoV-OC43, and HCoV-NL63 (provided by the National Clinical Testing Center) were selected. The nucleic acid was detected using the Yarui MA-6000 real-time PCR system.

#### 2.2.7 Clinical performance

Real-time SARS-CoV-2 Omicron assay clinical performance was evaluated using 456 known SARS-CoV-2 Omicron-positive clinical samples and 50 known SARS-CoV-2 Omicron-negative clinical samples in our hospital. The validation was conducted in our laboratory. The laboratory has obtained certification from the Zhejiang Clinical Laboratory Center (No. ZJ0153). All the reagents in the laboratory were approved by the national drug administration for In Vitro Diagnostics (IVDs), and their use was in accordance with Clinical Laboratory Improvement Amendments (CLIA) performance verification requirements to ensure the reproducibility of the detection performance as declared by the manufacturer.

#### 2.2.8 Statistical analysis

SPSS 20.0 (IBM, Armonk, NY, USA) and GraphPad Prism 9.0 (GraphPad, San Diego, CA, USA) were used for statistical analyses. The Mann-Whitney U test was used to compare two independent groups. The Kruskal-Wallis test and Dunn’s test were used to compare three independent groups. A p-value less than 0.05 is considered statistically significant. To assess the detection efficiency and diagnostic value of the RT-qPCR kits. The detection rate was determined using Pearson’s chi-square test.

#### 2.2.9 Ethical approval statement

This study was approved by scientific Research Ethics Committee of Hangzhou Hospital of Traditional Chinese Medicine (2023KLL101). The committee agreed on the clinical specimen data.

## 3. Results

### 3.1 Analysis of SARS-CoV-2 Omicron genotyping by mNGS

**Fig 1:**
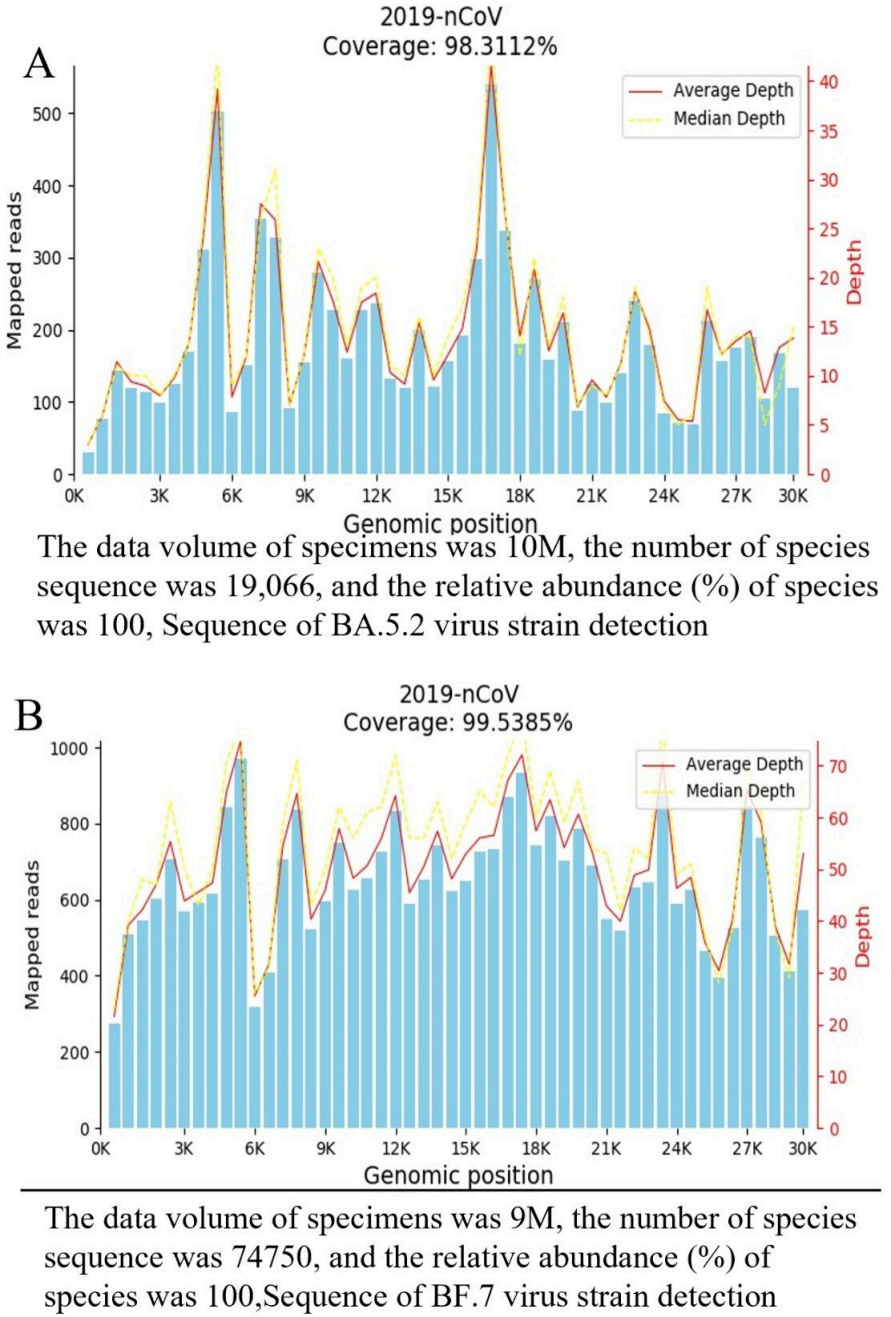
Microbial diversity associated with COVID-19 (A-B).

The average number of total reads was approximately 10 million and 9 million in the BA 5.2 and BF 7.0 for all the samples. Genotyping of throat swab and nasal swab specimens detected SARS-CoV-2 Omicron using mNGS technology. The detection coverage of 2019-nCoV was 98.31% in BA5.2 and 99.5385% in BF7.0 (Fig 1).

### 3.2 Coincidence rate verification

**Fig 2:**
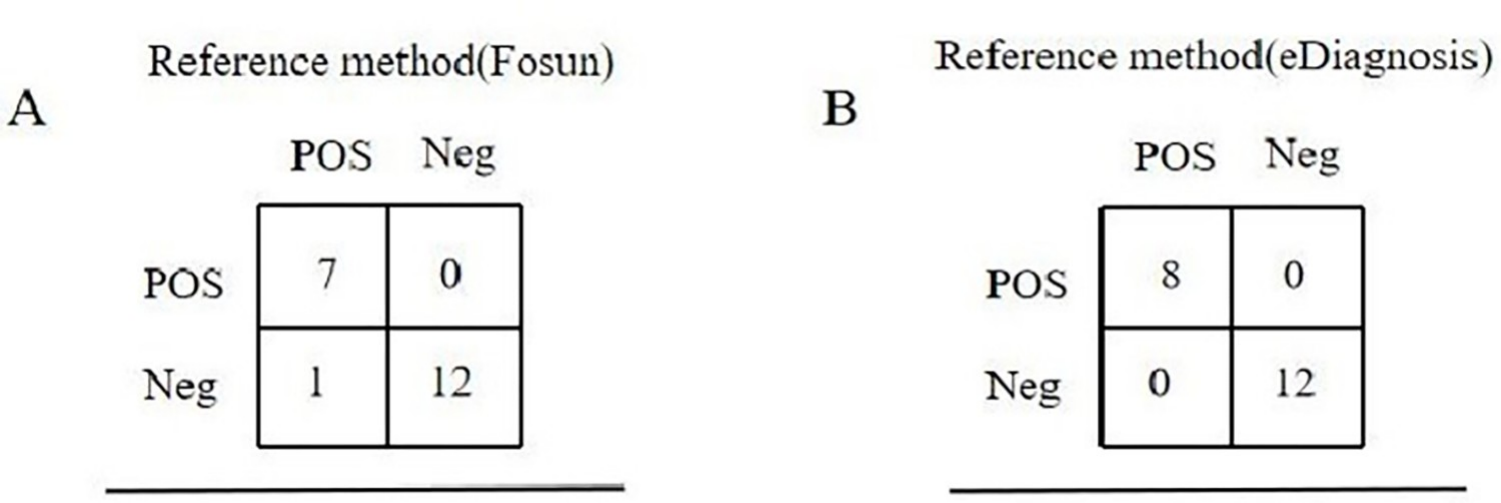
Coincidence rate of the two commercial RT-qPCR diagnostic test kits for SARS-CoV-2 Omicron evaluated in this study.

In 2022, there were 10 reference samples from the National Health Commission Clinical Laboratory Center and 10 patient samples from previous tests, totaling 20 samples. The coincidence rates for Fosun and eDiagnosis kits were 95% and 100%, respectively (Fig 2).

### 3.3 Analytical sensitivity of the novel SARS-CoV-2 Omicron real-time RT-qPCR assays

**Fig 3:**
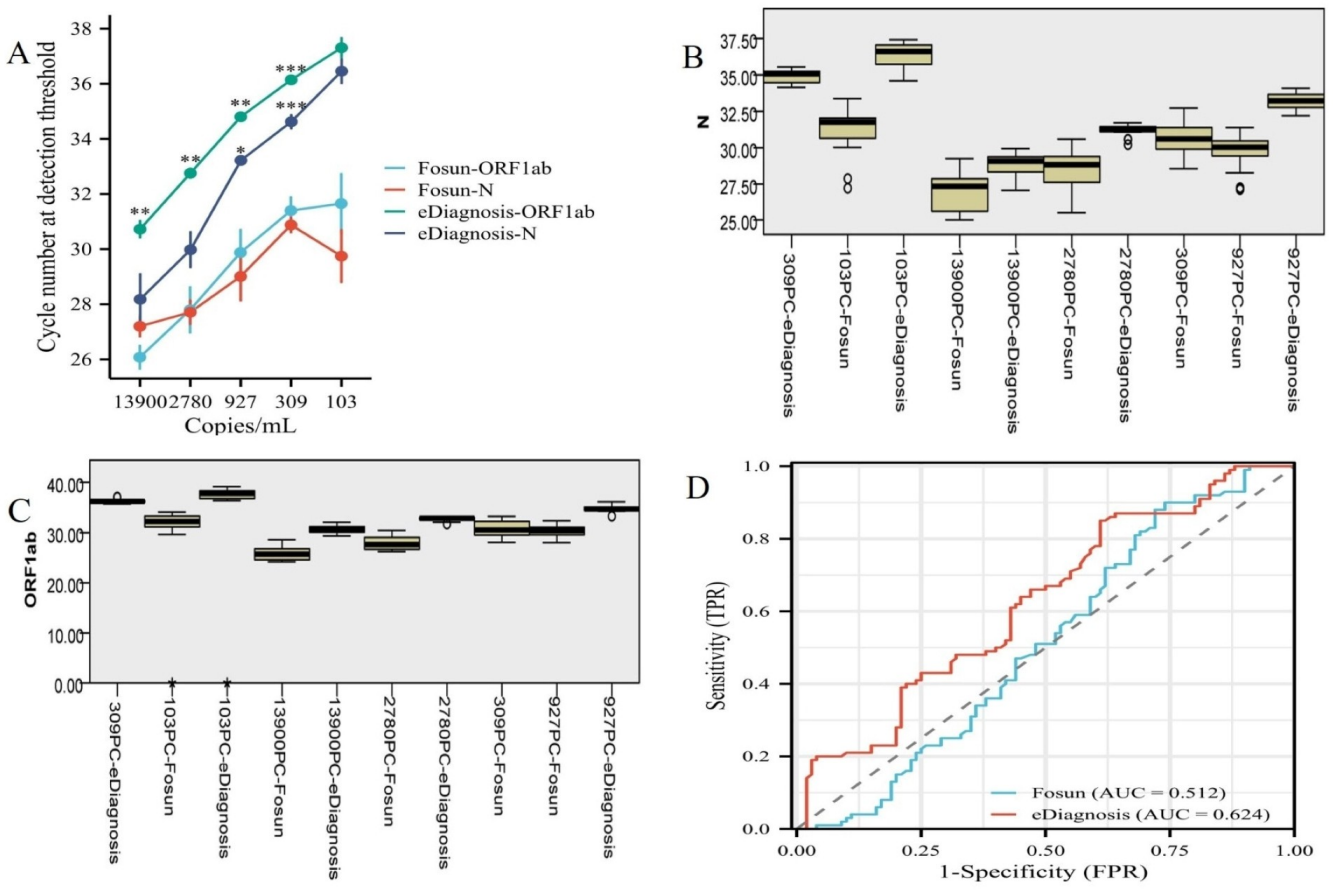
Analytical sensitivity of SARS-CoV-2 Omicron detected by real-time RT-qPCR.

Serial dilutions of SARS-CoV-2 Omicron culture isolate extracts were used to evaluate analytical sensitivity. In two independent amplification tests, respectively. The preliminary screening Limit of Detection (LoD) of Fosun was 300 copies/mL, whereas for eDiagnosis it was 500 copies/mL. The results showed that the mean thecycle number at detection threshold (*Cp*)values of ORF1ab and N genes were 31.40 and 30.88 for the Fosun kit, and 36.14 and 34.62 for the eDiagnosis kits (Fig 3A). From the LoD results, it was found that the thecycle number at detection threshold (*Cp*)value of Fosun kits was smaller than that of eDiagnosis kits. Therefore, we further studied the changes in the threshold (*Cp*) values of ORF1ab and N detected by the two kits at various concentrations. We observed that the thecycle number at detection threshold (*Cp*)value of the Fosun kits was lower than that of the eDiagnosis kit in detecting ORF1ab, showing significant statistical significance (P <0.01). Conversely, there was no significant difference between the Fosun and eDiagnosis kits at a concentration of 13900 PC (P > 0.05), while there was a statistically significant difference at other concentrations (Fig 3B and 3C). Despite variations in the C*p* values between the two kits, we assessed the diagnostic effectiveness of the reagents using the Receiver Operating Characteristic Curve (ROC) and Area Under ROC Curve, (AUC). The analysis revealed that the Receiver Operating Characteristic (ROC) of the eDiagnosis kits exceeded that of the Fosun kit (0.624 > 0.512), indicating that the eDiagnosis kit offers a greater advantage in clinical diagnostics (Fig 3D). Based on these results, we have determined that the thecycle number at detection threshold (*Cp*)value of the kit test results is directly linked to the design of the kit. Therefore, the clinical value of the kit cannot be assessed solely based on the *Cp* value of the test results. The Receiver Operating Characteristic (ROC) analysis showed that the ORF1ab and N assays of SARS-CoV-2 Omicron on the eDiagnosis platform yielded higher values compared to those on the Fosun platform.

### 3.4 Precision evaluation

**Fig 4:**
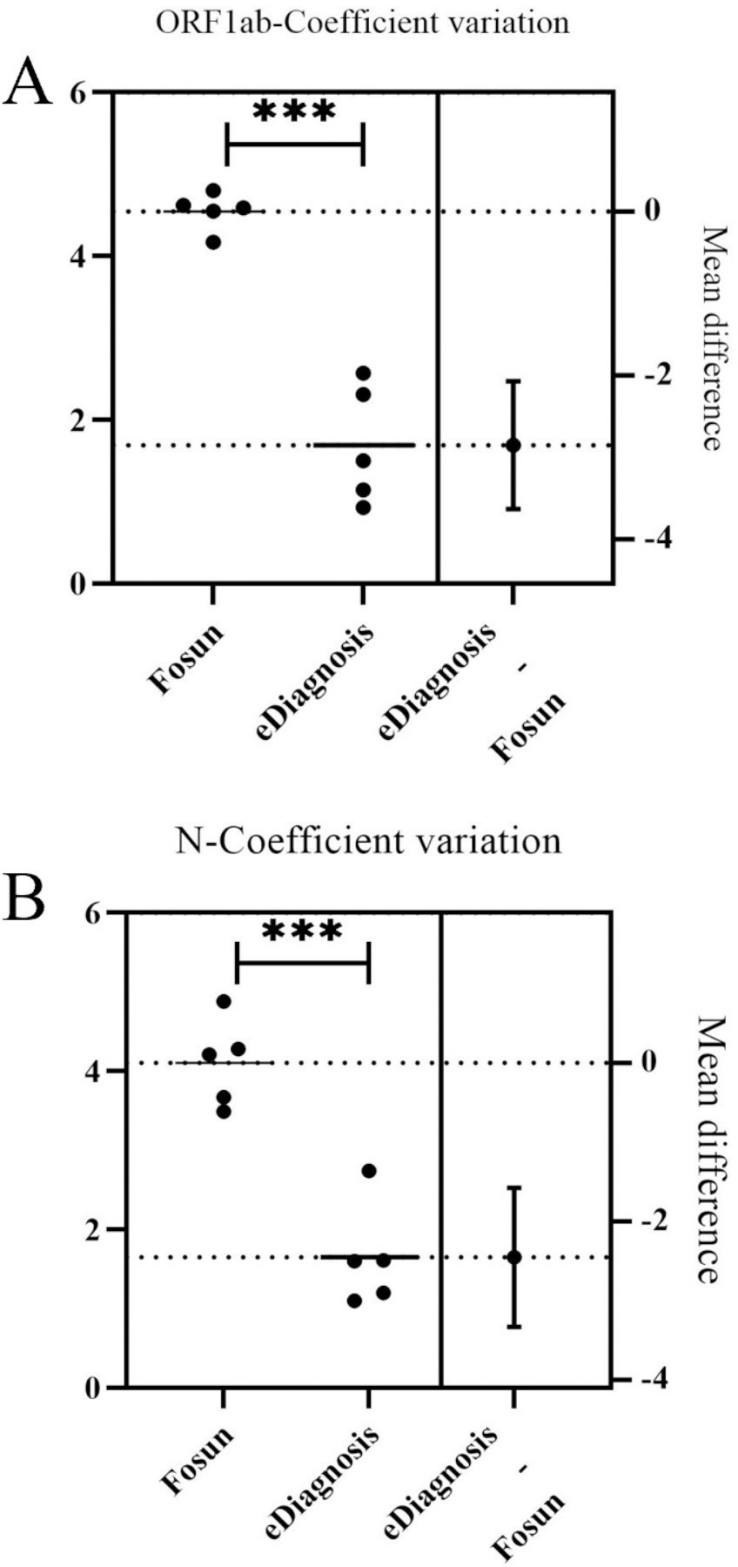
Precision results of the two commercial RT-qPCR diagnostic test kits for SARS-CoV-2 Omicron evaluated in this study.

The precision of the two commercial RT-qPCR diagnostic kits for SARS-CoV-2 Omicron was consistent with that declared by their respective manufacturers (coefficient of variation <5%, as shown in Table 2). The distribution of Cp values of 20 test results from each commercial RT-qPCR diagnostic test kit for SARS-CoV-2 Omicron is shown in Fig 4. As depicted, Fosun exhibited the highest coefficient of variation (CV) for ORF1ab gene detection above and below the detection line concentration (4.80%, Table 2), whereas eDiagnosis showed the lowest (0.93%, Table 2). The concentration of 103 copies/mL, however, had only 18 tests, of which two groups were not detected. Similarly, Fosun had the smallest coefficient of variation (CV) for the N gene at the detection limit concentration (3.49%, Table 2), while eDiagnosis exhibited an even smaller CV (1.10%, Table 2). At the same time, at a concentration of 10^3 copies/ml, the eDiagnosis assay only requires 18 tests, which is equivalent to the Fosun test. Taken together, our results indicate that eDiagnosis demonstrated optimal precision in detecting the ORF1ab and N genes at various concentrations.

**Table 2:** Fosun and eDiagnosis precision results of the five commercial RT-qPCR diagnostic test kits for SARS-CoV-2 Omicron evaluated in this study.

Concentrations	target genes	Mean (Fosun)	SD (Fosun)	CV% (Fosun)	Mean (eDiagnosis)	SD (eDiagnosis)	CV% (eDiagnosis)
13900copies/mL	ORF1ab	25.61	1.07	4.17	30.72	0.82	2.65
	N	27.03	1.32	4.88	28.85	0.79	2.74
	E	25.00	1.22	4.90			
2780 copies/mL	ORF1ab	27.81	1.28	4.62	32.75	0.38	1.15
	N	28.50	1.20	4.21	31.25	0.37	1.20
	E	28.00	1.40	5.00			
927 copies/mL	ORF1ab	30.25	1.39	4.59	34.75	0.55	1.50
	N	29.70	1.08	3.67	33.18	0.53	1.61
	E	29.00	1.14	3.96			
309 copies/mL	ORF1ab	30.79	1.48	4.80	36.23	0.33	0.93
	N	30.50	1.06	3.49	34.94	0.42	1.10
	E	30.13	0.95	3.19			
103 copies/mL	ORF1ab	32.07	1.46	4.55	37.83	0.87	2.31
	N	30.89	1.32	4.28	36.54	0.58	1.60
	E	30.65	1.26	4.11			

The coefficient of variation (CV) is calculated as a percentage of the standard deviation to the mean in the results.

### 3.5 Cross contamination and specificity

To investigate whether the novel Fosun and eDiagnosis assays cross-react with HCoV-NL63, HCoV-OC43, and other human-pathogenic coronaviruses, as well as respiratory viruses, the results showed that only the samples containing the SARS-CoV-2 Omicron virus (CVOP20S-01, 03, 06, 07, 08) were detected by the two detection reagents. The thecycle number at detection threshold (*Cp*) of the test results from both reagents met the criteria for positive results. Negative results (CVOP20S-02, 04, 05) were obtained in cross-reactivity tests, indicating that the two commercial RT-qPCR diagnostic tests for SARS-CoV-2 Omicron exhibited clear specificity. The presence of the listed pathogenic microorganisms did not affect the ability of the test kits to detect SARS-CoV-2 Omicron (Table 3).

**Table 3:** Pathogenic microorganisms used to evaluate the cross-reaction of two commercial RT-qPCR diagnostic test kits for SARS-CoV-2 Omicron evaluated in this study.

Sample code	Sample contenta	Viral RNAconcentrationt(ddPCRlog10 copies/mL)	Percentage correctqualitative results (all)		Reported Cqvalues (For information purposes only)		Laboratory reported results thecycle number at detection threshold (Cp) (Fosun and eDiagnostic)
			%	Total datasets	Median (range)	Total datasets	
CVOP20S-01	SARS-CoV-2	4.30	98.10	521.00	29.1 (15.8-41.6)	449.00	33.3/32.5
CVOP20S-02	HCoV-NL63	4.64	96.90	521.00	NA	NA	NA
CVOP20S-03	SARS-CoV-2	3.30	96.90	521.00	32.2 (18.0-43.0)	450.00	35.6/34.6
CVOP20S-04	HCOV-0C43	4.03	97.10	521.00	NA	NA	NA
CVOP205-05	Negative	NA	97.30	521.00	NA	NA	NA
CVOP20S-06	SARS-CoV-2	4.30	98.50	521.00	29.2 (16.6-40.0)	450.00	32.7/31.5
CVOP20S-07	SARS-CoV-2	5 30	99.20	521.00	25.9 (12.0-39.0)	453.00	34.2/28.7
CVOP20S-08	SARS-CoV-2	2.30	86.00	521.00	35.0 (22.7-43.3)	400.00	37.2/37.0

### 3.6 Clinical performance

**Fig 5:**
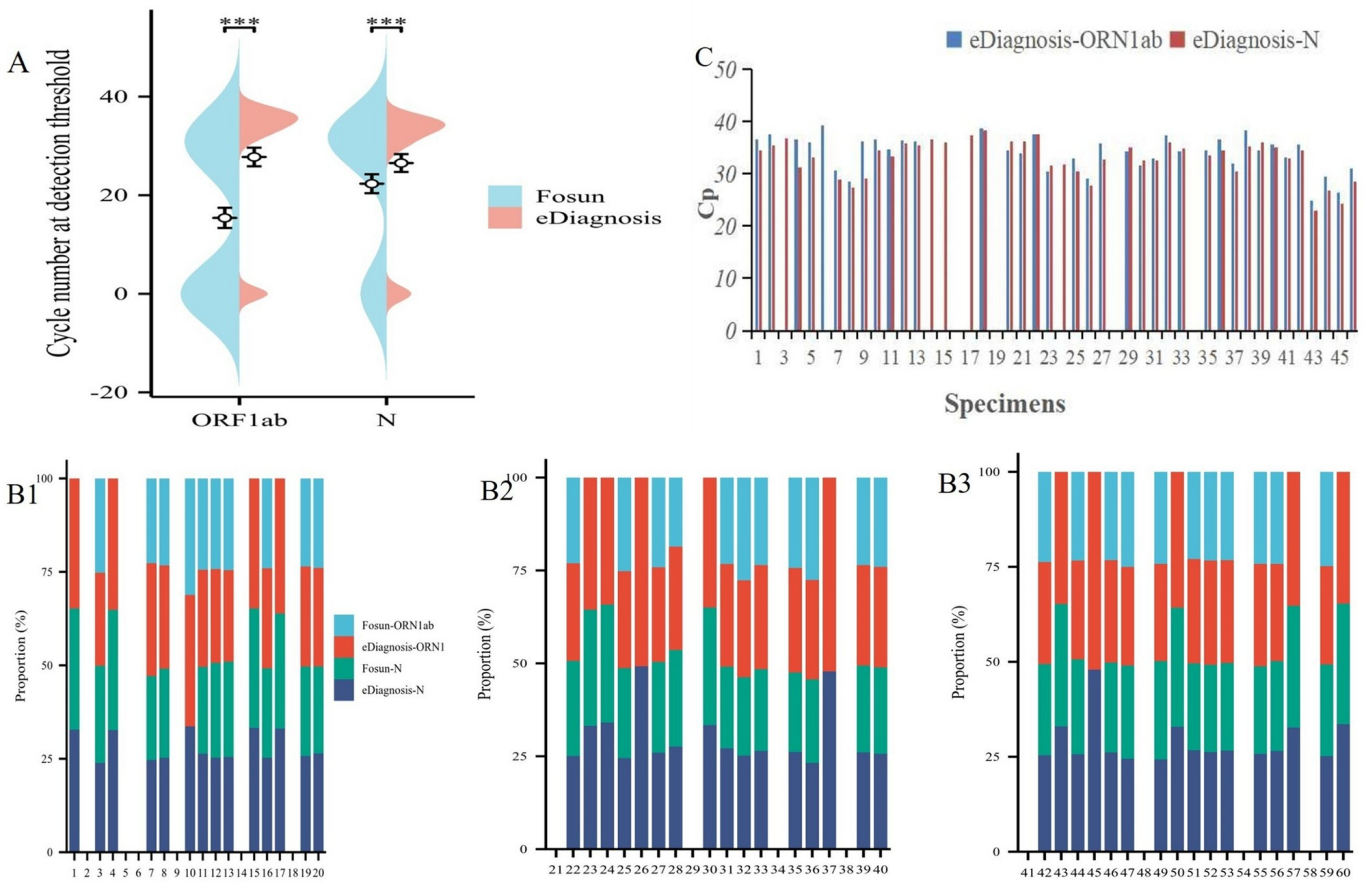
The number of reference product from Hangzhou Clinical Laboratory Center, that were positive for SARS-CoV-2 Omicron by the Fosun assay or eDiagnosis assay on different months includes 1-60 specimens.

Comparative performance of detecting SARS-CoV-2 Omicron in various clinical specimens. Firstly, clinical specimens were obtained from the Hangzhou Clinical Laboratory Center. We used the one-year reference product from Hangzhou Clinical Laboratory Center, which included 12 groups of reference products. Each group of quality control products consisted of 5 specimens with serial numbers ranging from 1 to 60. Through statistical analysis, the results indicated a significant difference in the detection of ORF1ab and N genes using the two reagents (P < 0.05) (Fig 5A). From 1 to 20, the results of positive samples were determined. It was further found that the Fosun reagents detected 10 out of 14 ORF1ab genes (71.2%) and 13 out of 14 N genes (92.8%), while the eDiagnosis reagents detected all 14 ORF1ab genes (100%) and all 14 N genes (100%). From 21 to 40, it was further found that the Fosun reagents detected 11 out of 16 (68.7%) ORF1ab genes and 14 out of 16 (87.5%) N genes, while the eDiagnosis reagents detected all 16 out of 16 (100%) ORF1ab genes and N genes. From 41 to 60, confirmed positive specimens showed that the Fosun tests detected ORF1ab and N genes in 68.7% (11/16) and 93.7% (15/16) of cases, respectively, while the eDiagnosis kits detected both genes in 100% (16/16) of cases (Fig 5B).

After reviewing all the results with reference product from the Hangzhou Clinical Laboratory Center, we determined that the sensitivity of the Fosun kit was 93.47% (43/46) and the specificity was 100% (14/14). The false-negative rate for the single-gene ORF1ab and N gene was 23.9% (11/46) and 4.3% (2/46), respectively, in the Fosun kit. However, the eDiagnosis kit demonstrates 100% sensitivity and specificity (46/46, 14/14). Through the validation of 12 batches of reference products, we have found that the sensitivity and specificity of eDiagnosis kits are more standardized. We confirmed that the nucleic acid extracted using the Fosun reagent was added simultaneously to the eDiagnosis mix system. The missed detection rate of ORF1ab and N gene was 15.2% (7/46) and 8.6% (4/46), respectively (Fig 5C). Upon verification, we have determined that the laboratory’s quality control involves a liquid matrix that may not align completely with Fosun’s extraction system, potentially resulting in a decrease in the purity of the extracted nucleic acid.

**Fig 6:**
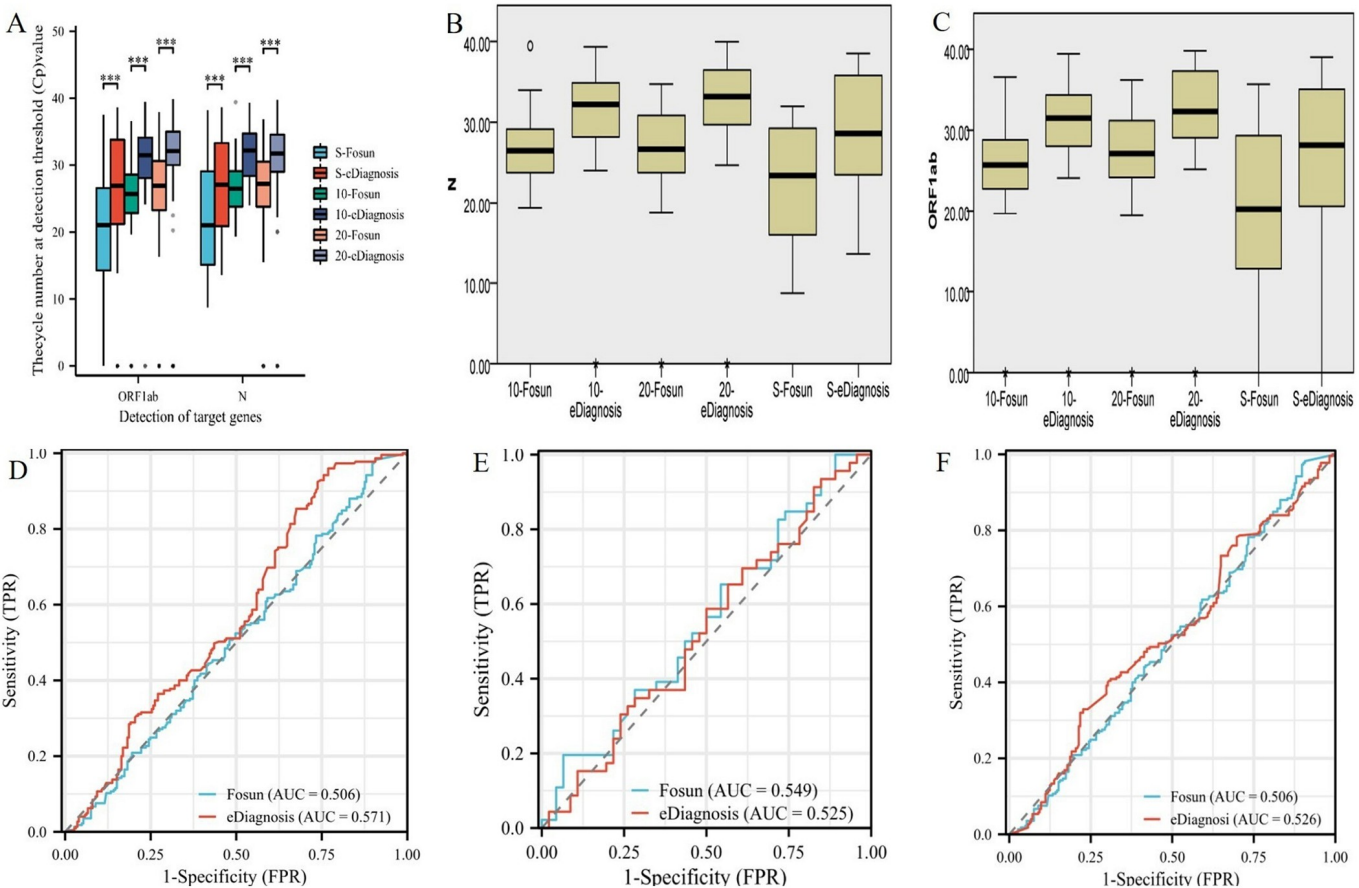
The number of clinical specimens that were positive for SARS-CoV-2 Omicron by the Fosun assay and eDiagnosis assay.

Secondly, we included samples of simple nasopharyngeal swabs (n = 183), 10-tube sampled specimens (n = 46), and 20-tube sampled specimens (n = 225), obtained from COVID-19 patients between December 2022 and January 2023. The results revealed a significant difference in the number of cycles of the detection threshold (Cp) value, as measured by the concentration of each nucleic acid reference substance in each group (P < 0.01). The *Cp* of the eDiagnosis reagent was significantly higher than that of the Fosun reagent (Fig 6A–6C). The validation of clinical specimens and the performance verification of the previous reagents have been consistent. This also complies with the relevant kit instructions.

Through the verification of a large number of clinical specimens, it was found that there were missed detection events of single-target genes with the two reagents in the S group. There was a significant difference in the ORF1ab gene of the two reagents only in the apheresis specimens (P < 0.01) (Table 4). The percentage of positive single target genes detected for the ORF1ab gene of Fosun was 10.9% (20/183), while the percentage of positive single target genes detected in the ORF1ab gene of eDiagnosis was 3.2% (6/183). However, the sensitivity and specificity of both eDiagnosis and Fosun reagents are equal. There was no significant difference in the clinical diagnostic efficiency of the two reagents (Fig 6D–6F).

**Table 4:** Comparison of the non-detection rate and detection rate of the single-target ORF1ab gene using Fosun and eDiagnosis kits in SARS-CoV-2 Omicron.

Group	Undetected rate(%)	Detection rate(%)	P
S- Fosun	10.9	89.1	<0.01
S-eDiagnosis	3.2	96.8	
10- Fosun	10.9	89.1	0.06
10-eDiagnosis	4.3	95.7	
20- Fosun	9.3	90.7	0.4
20-eDiagnosis	5.7	94.3	

The detection rate was determined using Pearson’s chi-square test. The dataset consistently comprised 200 data points, with no cells (0%) having an expected count of less than 5. The minimum expected count is 7.50.

Among the 10-tube sampled specimens, it was determined that out of 46 positive specimens, the percentage of positive single target genes detected for the ORF1ab gene of Fosun was 10.8% (5/46), while the percentage of positive single target genes detected in the ORF1ab gene of eDiagnosis was 4.3% (2/46). The sensitivity and specificity of both eDiagnosis and Fosun reagents are equal (100%), and there was no significant difference in the comparison of the other groups (Table 4).

Among the 20 tube-sampled specimens, it was determined that out of 225 positive specimens, the percentage of positive single target genes detected for the ORF1ab, N, and E genes of Fosun was 9.33% (21/225), 1.77% (4/225), and 1.33% (3/225). The failure detection rate was 1.77% (4/225). While the number of positive single-target genes detected in the ORF1ab and N gene of eDiagnosis was 5.77% (13/225) and 1.77% (4/225). The non-detection rate was 1.33 (3/225). The sensitivity and specificity of both eDiagnosis and Fosun reagents are equal (98.7%/100%), and there was no significant difference in the comparison of the other groups (Table 4).

## 4. Discussion

In our evaluation, we utilized the SARS-CoV-2 Omicron liquid performance verification reference product in conjunction with clinical diagnostic efficacy to independently and comprehensively validate the performance of two commercially available SARS-CoV-2 Omicron RT-qPCR diagnostic kits that are currently being utilized in our local laboratory [16, 17]. As described in the Results section, we estimated the Limit of Detection (LoD) of the SARS-CoV-2 Omicron Nucleic Acid Test Kit (Fosun) to be approximately 300 viral RNA copies/mL of the sample, which aligns with the LoD specified in the manufacturer’s manual. Moreover, we estimated the LoD of the SARS-CoV-2 Omicron Nucleic Acid Diagnostic Kit (eDiagnosis) to be 500 viral RNA copies/mL of the sample, slightly higher than the 300 copies/mL LoD required in the Fosun kit (Fig 3A). However, the LoD values provided by both kits are entirely acceptable for a reliable diagnosis of SARS-CoV-2 Omicron, considering the frequency distributions of viral loads in the population [18, 19]. On Table 2, analytical parameters and other characteristics for SARS-CoV-2 Omicron Fosun and eDiagnosis are summarized. Results analyses showed that Fosun had a higher coefficient of variation (CV) for detecting ORF1ab and N genes both above and below the detection line concentration, whereas eDiagnosis had a lower CV. These results demonstrate that the stability and repeatability of the eDiagnosis reagent were relatively better, as illustrated in (Fig 3D). Analytical sensitivity and precision for SARS-CoV-2 Omicron detection is a crucial performance metric for evaluating viral detection assays. Analytical sensitivity and precision for virus detection is a significant factor in selecting an assay to use, but it is not the only consideration. The sensitivity precision of the test may be influenced by the specimen matrix, which can impact the ability to detect the virus or its compatibility with different types of specimens. This is especially important for methods that skip the extraction step and allow for the direct analysis of original swab samples [3, 5, 20].

Additionally, we observed that different kits exhibited varying levels of sensitivity to the ORF1ab and N genes. Specifically, Fosun and eDiagnosis kits were more sensitive in detecting the N gene compared to the ORF1ab gene. The reagent company was selected based on the characteristics of SARS-CoV-2 Omicron [21, 22]. The two commercial RT-qPCR diagnostic tests for SARS-CoV-2 Omicron demonstrated clear specificity. The presence of other pathogenic microorganisms did not affect the ability of the test kits to detect SARS-CoV-2 Omicron (Table 3). Through testing two reagents, the performance verification results for the qualitative aspects of SARS-CoV-2 Omicron meet the requirements outlined in their respective instructions.

In addition, we used quality control samples from the Hangzhou Provisional Inspection Center to validate the performance of the two reagents. The findings suggest that there was a difference *Cp* value in the detection of ORF1ab and N genes using the two reagents (Fig 5A and 5B). This difference is statistically significant (p < 0.05). When we conducted the data statistics, the Fosun reagent exhibited a significantly higher detection rate for a single target gene compared to the eDiagnosis reagent. This was particularly evident in samples with low concentrations of the SARS-CoV-2 Omicron variant, leading to a significant number of missed detections during specimen testing. The missed detection rate is as high as 23.9% in reference product from Hangzhou Clinical Laboratory Center. After reviewing all the results with the testing center, we determined that the sensitivity of the Fosun kit was 93.47% (43/46) and the specificity was 100% (14/14). However, the eDiagnosis kit demonstrates 100% sensitivity and specificity (46/46, 14/14). Further investigation revealed that the liquid substance provided as quality control by Hangzhou Clinical Laboratory Center may have an impact on Fosun’s extraction system. This could potentially lead to insufficient purity in the extraction process and increase the risk of undetected samples [23, 24].

All variants of COVID-19 can cause severe illness or death, especially in the most vulnerable populations, so prevention is always critical [20, 21]. The diffusion of volatile organic compounds depends on preventive measures, such as ventilation. The number of infections will depend on the extent to which this variant evades the protection of the vaccine, as well as the effectiveness of enhancing the vaccine in boosting immunity. Unfortunately, both factors remain unclear, and vaccines alone may not be potent enough to control the pandemic [22]. Therefore, the most effective method is to detect positive patients using RT-qPCR technology and isolate infected individuals promptly. Sequencing analyses have shown that the Fosun test for SARS-CoV-2 Omicron detection is sensitive (87.5%) and specific (100%). eDiagnosis tests for SARS-CoV-2 Omicron detection are highly sensitive (100%) and specific (100%) when screening respiratory tract samples from COVID-19 patients. Through the system verification of the two reagents for the positive samples, it was found that the target failure detection rate of the Fosun reagent was higher than that of the eDiagnosis reagent, reaching 1.77%. The detection rate of individual genes, especially the ORF1ab gene, was higher when using the eDiagnosis reagent compared to the F reagent. Both reagents successfully detected the N gene. This situation is completely consistent with the verification provided earlier.

Here, one of our limitations is our ability to only provide a concise comparison of the assay performance of the kit, which may not fully represent the assay performance of other batches of kits. Another limitation is the inconsistency in the number of positive and negative specimens in the three groups of samples during the validation of clinical specimens. This inconsistency is primarily attributed to the rapid spread of the virus at that time. According to CDC requirements, testing should be conducted based on the principle of voluntary participation [8]. Suspected individuals should be promptly tested, identified, reported, and managed. To enhance efficiency, more than the 20 tube-sampled specimens were used for screening. Apheresis is performed on individuals under suspicion, leading to variations in the number of specimens collected. In summary, we evaluated the performance of two commercial RT-qPCR diagnostic assays for detecting SARS-CoV-2 Omicron and provided recommendations for selecting kits for clinical laboratories to enhance the commercial optimization of RT-qPCR diagnostic assays. These measures should reduce the clinical risks associated with false-negative results and enhance the detection and control of the spread of COVID-19 more effectively worldwide. The variation and spread of SARS-CoV-2 Omicron in multiple geographic locations suggest the necessity of regular genetic screening. In our future work, we plan to evaluate the genetic changes in the virus and their impact on detection using existing commercial kits for SARS-CoV-2 Omicron [25].

## 5. Conclusion

During the validation of the BSD quality control, it was observed that both reagents met the requirements outlined in their respective instructions. The special feature of the Fosun reagent is that it targets a single gene (ORF1ab, N, E). The ORF1ab and N assays of SARS-CoV-2 Omicron on the eDiagnosis platform yielded higher values compared to those on the Fosun platform. Consequently, the eDiagnosis kit has also been used as standard detection reagents. According to CDC requirements, testing for SARS-CoV-2 Omicron requires the use of two reagents for detection and confirmation. Given that the Fosun reagent has a relatively low detection limit and targets three single genes, it is more advantageous as a confirmatory reagent for the new virus strain.

## Supporting information

S1 Data Graphs and tables for the overall total data. (XLSX)

## Abbreviations

mNGS: Metagenomic Next-Generation Sequencing
RT-qPCR: Real-Time quantitative PCR
CV: coefficient of variation
ORF: The open reading frame
LOD: Limit of Detection
Cp: detection threshold
ROC: Receiver Operating Characteristic Curve
AUC: Area Under ROC Curve
